# Association of Circulating Vitamin E (α- and γ-Tocopherol) Levels with Gallstone Disease

**DOI:** 10.3390/nu10020133

**Published:** 2018-01-27

**Authors:** Sabina Waniek, Romina di Giuseppe, Tuba Esatbeyoglu, Ilka Ratjen, Janna Enderle, Gunnar Jacobs, Ute Nöthlings, Manja Koch, Sabrina Schlesinger, Gerald Rimbach, Wolfgang Lieb

**Affiliations:** 1 Institute of Epidemiology, University of Kiel, 24105 Kiel, Germany; sabina.waniek@epi.uni-kiel.de (S.W.); romina.digiuseppe@epi.uni-kiel.de (R.d.G.); ilka.ratjen@epi.uni-kiel.de (I.R.); janna.enderle@epi.uni-kiel.de (J.E.); jacobs@popgen.de (G.J.); mkoch@hsph.harvard.edu (M.K.); 2 Institute of Human Nutrition and Food Science, University of Kiel, 24118 Kiel, Germany; tuba.esatbeyoglu@mri.bund.de (T.E.); rimbach@foodsci.uni-kiel.de (G.R.); 3 Biobank PopGen, University Hospital Schleswig-Holstein, Campus Kiel, 24105 Kiel, Germany; 4 Department of Nutrition and Food Science, University of Bonn, 53113 Bonn, Germany; noethlings@uni-bonn.de; 5 Department of Nutrition, Harvard T.H. Chan School of Public Health, Boston, MA 02115, USA; 6 Institute for Biometrics and Epidemiology, German Diabetes Center (DDZ) at Heinrich Heine University Duesseldorf, 40225 Duesseldorf, Germany; sabrina.schlesinger@DDZ.uni-duesseldorf.de

**Keywords:** vitamin E, α- and γ-tocopherol, gallstone disease

## Abstract

In addition to well-established risk factors like older age, female gender, and adiposity, oxidative stress may play a role in the pathophysiology of gallstone disease. Since vitamin E exerts important anti-oxidative functions, we hypothesized that circulating vitamin E levels might be inversely associated with prevalence of gallstone disease. In a cross-sectional study, we measured plasma levels of α- and γ-tocopherol using high performance liquid chromatography in a community-based sample (582 individuals; median age 62 years; 38.5% women). Gallstone disease status was assessed by ultrasound. Multivariable-adjusted logistic regression models were used to estimate the association of circulating α- and γ-tocopherol/cholesterol ratio levels with prevalent gallstone disease. Lower probabilities of having gallstone disease were observed in the top (compared to the bottom) tertile of the plasma α-tocopherol/cholesterol ratio in multivariable-adjusted models (OR (Odds Ratio): 0.31; 95% CI (Confidence Interval): 0.13–0.76). A lower probability of having gallstone disease was also observed for the γ-tocopherol/cholesterol ratio, though the association did not reach statistical significance (OR: 0.77; 95% CI: 0.35–1.69 for 3rd vs 1st tertile). In conclusion, our observations are consistent with the concept that higher vitamin E levels might protect from gallstone disease, a premise that needs to be further addressed in longitudinal studies.

## 1. Introduction

In addition to well-established risk factors such as age, female gender, obesity, and dyslipidemia [1,2,3], oxidative stress is thought to play a role in the pathophysiology of gallstone disease [4,5,6]. Vitamin E is a lipid-soluble vitamin encompassing different tocopherols (α-, β-, γ-, and δ-tocopherol) and tocotrienols (α-, β-, γ-, and δ-tocotrienol) with important anti-oxidative functions [7], and could, therefore, potentially affect the risk to develop gallstones. Indeed, in prior, rather small studies, α-tocopherol levels [8] and the α-tocopherol/cholesterol ratio [9,10] were found to be lower in individuals with gallstone disease (*n* = 16–30) compared to healthy controls (*n* = 20–47) [8,9,10]. While these results are intriguing, population-based analyses regarding the association of vitamin E levels (including both α- and γ-tocopherol) with gallstone disease in larger samples are currently lacking.

Considering that α- and γ-tocopherol are the two major vitamers in the human body [11], and that more than 90% of the German population consume vitamin E in the form of α- and γ-tocopherol [12], we aimed to investigate cross-sectional associations of both α- and γ-tocopherol plasma levels with gallstone disease in a community-based sample from Northern Germany.

## 2. Subjects and Methods

### 2.1. Study Design and Sample

The study sample comprised 1316 individuals from Northern Germany, recruited by the PopGen biobank in Kiel, Germany. Of them, 747 participants were derived from a random sample of the general population of Kiel, and 569 individuals were blood donors recruited at the University Hospital Schleswig-Holstein in Kiel (Germany), between 2005 and 2007 [13]. A total of 952 individuals agreed to participate in the first follow-up examination (between 2010 and 2012) where blood sampling and a medical examination was performed. Furthermore, participants filled-out standardized questionnaires on demographics and various health-related characteristics, including smoking status, medical history, dietary intake, and physical activity (please see below for details) [13,14,15]. Ultrasound examinations of the upper abdomen were performed in a subsample of 846 participants (please see below for details). Participants with missing information on ultrasound examination (*n* = 4) or diagnosis of gallbladder sludge (*n* = 6) were excluded, leaving 836 participants with ultrasound information regarding gallstone disease. In 582 of these participants, circulating vitamin E (α- and γ-tocopherol) concentrations were available. The study was approved by the Ethics Committee of the Medical Faculty of the Christian-Albrechts University of Kiel (Project identification code A 156/03). All participants provided written informed consent.

### 2.2. Ultrasound Examination

Gallstone disease was defined as gallbladder stones visualized at the ultrasound examination. To this end, an ultrasound examination of the upper abdomen was conducted under standard conditions using a Logiq 400 cl real-time ultrasound system (General Electric Healthcare, Bedford, UK) with a convex array probe (3-Mhz). Ultrasound examinations were carried out in supine position, the right arm raised over the head, and was performed under deep inspiration. The gallbladder was examined in 3 planes (longitudinal, cross-sectional, and diagonal) to provide the examiner with a 3-dimensional impression of the organ. In cases in which gallstones were present, the mobility of the stone(s) was assessed to differentiate gallstones from gallbladder polyps. Biliary sludge was identified as low-amplitude echoes without acoustic shadowing.

### 2.3. Physical Examinations and Standardized Questionnaires

Weight and height were measured in light clothing without shoes. Two kilograms was subtracted from weight measurements to account for clothing. Body mass index (BMI) was calculated as body weight (kg) divided by height squared (m^2^). Waist circumference was measured at the midpoint between the lower ribs and iliac crest. After the participants had rested at least 5 min in a sitting position, blood pressure was measured two times at the right arm using a sphygmomanometer [16], and the arithmetic mean of both measurements was used for our analyses. Information on dietary intake, including vitamin E supplementation, and alcohol consumption during the past 12 months, were assessed using a self-administered semi-quantitative 112-item food-frequency questionnaire (FFQ), designed and validated for the German population [14]. Total energy intakes were determined using the German Food Code and Nutrient Data Base (version II.3), and were provided by the Department of Epidemiology of the German Institute of Human Nutrition Potsdam Rehbrücke [17].

### 2.4. Definition of Covariates Assessment

Prevalent hypertension was defined as systolic blood pressure ≥140 mmHg, or diastolic blood pressure ≥90 mmHg, or use of antihypertensive medication, or self-reported hypertension history. Type 2 diabetes was defined as use of anti-diabetic medication or glycated hemoglobin (HbA1c) ≥6.5% (48 mmol/mol) or fasting serum glucose ≥126 mg/dL, or self-reported physician diagnosis. Liver disease was defined as self-reported hepatitis A, B, C, or D virus infection, hemochromatosis, autoimmune liver disease, or liver cirrhosis diagnosis.

In order to quantify the amount of physical activity, participants were asked to report their time spent walking, cycling, “do-it-yourself” activities, gardening, sports, and household chores during the past 12 months, and the average number of stairs climbed per day [18]. The duration of each physical activity was then multiplied by the corresponding metabolic equivalent task (MET)-values and summed over all activities [19,20]. To determine smoking status, participants were classified into three categories: no-smokers (never smoked), former smokers (smoked in the past and quit smoking more than 1 year ago), and current smokers (currently smoking 1 or more cigarettes per day). According to their level of education, participants were categorized into three categories: low (≤9 years), middle (10 years), or high (≥11 years).

### 2.5. Biochemical Measurements

Blood samples were obtained from participants in a sitting position after overnight fasting. HbA1c, glucose, triglycerides, HDL-cholesterol, and low density lipoprotein (LDL)-cholesterol and total cholesterol were determined in fresh blood samples under standard conditions on the same day in the Institute of Clinical Chemistry at the University Hospital Schleswig-Holstein, Campus Kiel. The Institute of Human Nutrition and Food Science from the University of Kiel (Germany) measured plasma vitamin E (α- and γ-tocopherol) levels using high performance liquid chromatography (HPLC) with fluorescence detection. The methods have been described in detail elsewhere [21], with the coefficients of variation for α- and γ-tocopherol being 1.09% and 1.29%, respectively.

### 2.6. Statistical Analyses

Missing covariate values (*n* = 13) were imputed as follows: missing categorical variables were imputed by the most commonly observed category of that respective variable (*n* = 9), and missing values of normally distributed continuous variables were replaced by the respective mean, whereas skewed variables were imputed by the sex-specific median (*n* = 4). When values of γ-tocopherol were below the detection limit (*n* = 14), they were imputed by the lowest γ-tocopherol concentration measured in our study sample. Because vitamin E circulates in the blood bound to lipoproteins [22,23], circulating vitamin E (α- and γ-tocopherol) levels (μmol/L) were divided by circulating total cholesterol levels (mmol/L), and used as α-tocopherol/total cholesterol ratio (μmol/mmol) and γ-tocopherol/total cholesterol ratio (μmol/mmol) as primary exposure variables in the statistical analyses.

The following analyses were performed: first, for descriptive purposes, differences in the characteristics of the participants with and without gallstone disease were tested for statistical significance using the chi-squared test or the Fisher’s exact test, as appropriate, for categorical variables, and the Wilcoxon’s rank-sum test for continuous variables. Second, logistic regression models were conducted to estimate the associations of the α- and γ-tocopherol/cholesterol ratio (each ratio considered separately and modeled in tertiles) with gallstone disease. Age- and sex-adjusted, as well as multivariable-adjusted logistic regression models, were performed. The multivariable-adjusted model controlled for age (continuous in years), sex (female, male), education (low, medium, high), physical activity (continuous in MET-hour/week), smoking status (never, current, former), vitamin E supplementation (yes, no), alcohol intake (continuous in g/day), total energy intake (continuous in kJ/day), and BMI (continuous in kg/m^2^). Categorical variables with more than 2 categories were included as indicator variables.

Third, we performed several sensitivity analyses: we repeated the above mentioned logistic regression models, after (a) excluding vitamin E supplement users (*n* = 44, prevalent gallstone cases = 3); (b) excluding individuals with self-reported liver disease (hepatitis A, B, C, or D virus infection, hemochromatosis, autoimmune liver disease, or liver cirrhosis) (*n* = 30, prevalent gallstone cases = 1) from our analyses; and (c) excluding individuals with type 2 diabetes (*n* = 61, prevalent gallstone cases = 7). In further sensitivity analyses, we used α- and γ-tocopherol (without adjustment for cholesterol) as the exposure variables, and assessed the associations of these biomarkers with gallstone disease. Finally, we excluded all individuals with any missing values, and performed a complete case analysis (*n* = 558, prevalent gallstone cases = 45).

Analyses were performed with SAS 9.4 (SAS Institute, Cary, NC, USA). Statistical tests were 2-sided, and *p* values <0.05 were considered statistically significant.

## 3. Results

### 3.1. General Characteristics

Overall, 46 individuals (7.9%) of the study sample had gallstones detected by ultrasound. Participants with gallstones were older and had lower circulating α-tocopherol and α-tocopherol/cholesterol ratio levels compared to participants without gallstones. A total of 44 participants (7.6%) of the study sample were taking vitamin E supplements (Table 1).

### 3.2. Association of α- and γ-Tocopherol/Cholesterol Ratio with Gallstone Disease

Lower probabilities of having gallstone disease were observed in the top tertile compared to the bottom tertile of the plasma α-tocopherol/cholesterol ratio in both age- and sex-adjusted (OR: 0.32; 95% CI: 0.13–0.78), as well as in multivariable-adjusted models (OR: 0.31; 95% CI: 0.13–0.76). Likewise, a lower probability of having gallstones for the γ-tocopherol/cholesterol ratio was observed in the top as compared to the bottom tertile, but this association did not reach statistical significance (Table 2).

### 3.3. Sensitivity Analyses

The associations between α- and γ-tocopherol/cholesterol ratio and gallstone disease did not substantially change in the sensitivity analyses after (a) excluding vitamin E supplement users (*n* = 44); (b) excluding individuals with self-reported liver disease (*n* = 30); and (c) excluding individuals with type 2 diabetes (*n* = 61). Likewise, the results were similar when we modeled both α- and γ-tocopherol as exposure variables, without dividing them by cholesterol, and when we performed a complete case analysis (*n* = 558) (Supplementary Table S1).

## 4. Discussion

### 4.1. Principle Observations

We examined the cross-sectional association of circulating vitamin E (α- and γ-tocopherol) levels with ultrasound-detected gallstone disease in a population-based sample from Northern Germany. Our main observations were as follows: first, the prevalence of gallstone disease in our sample was 7.9%. Second, participants with gallstone disease had lower circulating α-tocopherol and α-tocopherol/cholesterol ratio levels compared to participants without gallstone disease, and this inverse association of the α-tocopherol/cholesterol ratio with gallstone disease remained statistically significant in multivariable-adjusted models.

### 4.2. In the Context of the Current Literature

#### 4.2.1. Prevalence of Gallstone Disease

The prevalence of gallstone disease in our sample (7.9%) is slightly higher compared to other German studies, likewise detecting gallstones by ultrasound (3.9%, *n* = 1116 [24] and 4.1%, *n* = 2147 [25], respectively). However, the prevalence of gallstone disease in our study sample is lower than in the Study of Health in Pomerania (SHIP), a community-based sample from North-East Germany (*n* = 4202), where a slightly higher prevalence (10.1%) of gallstone disease was reported [26]. Yet, the region where the SHIP study is conducted is known for its strong clustering of metabolic risk factors [27]. Similar to our observations, data from the National Health and Nutrition Examination Survey III (*n* = 14,238), collecting nationally representative data from the population of the United States, documented a prevalence of gallstone disease of about 8.1% [28].

#### 4.2.2. Vitamin E (α- and γ-Tocopherol) Levels and Gallstone Disease

Our data suggest that higher circulating vitamin E levels are associated with a lower probability of having gallstone disease. This is in line with some prior observations, obtained in clinical settings on rather smaller samples [8,9,10].

Rocchi et al. [10] observed lower levels of the plasma α-tocopherol/cholesterol ratio in patients with gallstone disease (*n* = 16) compared to healthy controls (*n* = 20), and Shukla et al. [8] reported lower levels of serum α-tocopherol in 30 individuals with gallstone disease compared to 30 healthy controls. Consistently, Worthington et al. [9] reported lower levels of the serum α-tocopherol/cholesterol ratio in 18 patients with (ultrasound detected) gallstone disease, compared to 47 healthy controls. We extend these prior observations by demonstrating an inverse association of higher α-tocopherol/cholesterol ratio levels with gallstone disease in a larger, community-based sample, including 46 participants with ultrasound evidence of gallstone disease and 536 individuals where gallstones had been ruled out by ultrasound.

### 4.3. Potential Mechanisms for the Observed Association

Free radicals and oxidative stress may play a role in the pathophysiology of gallstone disease [6]. In agreement with this concept, higher levels of oxidative stress markers have been found locally in the gallbladder mucosa as well as in the circulation of patients with gallbladder disease as compared to individuals free of gallbladder disease [4,5].

Thus, one potential explanation for the observed inverse association between circulating plasma α-tocopherol levels and gallstone disease would be that individuals with higher vitamin E levels are better protected from oxidative stress and, therefore, have a lower probability to develop gallstones. However, due to the cross-sectional design of our analyses, we cannot rule out reverse causality, which would mean that the lower vitamin E levels observed in individuals with gallstone disease could be secondary to increased oxidative stress in those with gallstone disease.

Furthermore, bile acids are synthesized in the liver and secreted into the intestine, where they emulsify dietary lipids, including fat-soluble vitamins [29]. Some reports indicated impaired vitamin E absorption under cholestatic conditions [30,31]. It has been shown that disturbed bile secretion due to cholestatic diseases is associated with lipid-soluble vitamin deficiencies [32].

### 4.4. Strength and Limitations

Strengths of the present study include the moderate-sized population-based sample, the measurement of plasma vitamin E (α- and γ-tocopherol) levels, and the detailed and standardized assessment of covariates. The following limitations merit consideration: the cross-sectional design precludes causal inferences, because exposure and outcome were assessed at the same time point. Besides, the present cohort is not representative of the general population, due to the inclusion of a subgroup of blood donors. However, considering our research question, this limitation could play a limited role with respect to the overall research findings. Lastly, we cannot completely rule out model misspecification, though we identified important covariates affecting both exposures and outcome. Therefore, we built parsimonious models incrementally adjusted for covariates to provide the reader with a comprehensive picture.

## 5. Conclusions

In conclusion, we observed an inverse association between circulating vitamin E (α- and γ-tocopherol) levels and the prevalence of gallstone disease in a community-based sample. This observation supports the concept that higher levels of the antioxidant vitamer α-tocopherol may protect against gallstone disease, a premise that needs to be addressed in a prospective setting. Furthermore, it needs to be established if, and to what, extent dietary vitamin E supplementation may prevent gallstone formation.

## Figures and Tables

**Table 1:** General characteristics of the PopGen control study population by gallstone disease status (*n* = 582).

Clinical Features	All (*n* = 582)	Gallstones, Yes (*n* = 46)	Gallstones, No (*n* = 536)	*p*
Men, %	61.5	54.4	62.1	0.273
Age, years	62.0 (55.0, 70.0)	67.0 (60.0, 73.0)	62.0 (54.0, 70.0)	0.019
Body mass index, kg/m^2^	26.6 (24.2, 29.3)	27.4 (25.2, 29.8)	26.5 (24.1, 29.3)	0.292
Waist circumference, cm	96.0 (87.9, 104.2)	96.9 (83.7, 105.8)	95.9 (88.3, 104.2)	0.659
Systolic blood pressure, mmHg	138.5 (125.0, 150.0)	135.0 (122.5, 145.0)	138.8 (125.0, 150.0)	0.277
Diastolic blood pressure, mmHg	85.0 (80.0, 90.0)	83.8 (80.0, 90.0)	85.0 (80.0, 90.0)	0.736
Prevalent hypertension, %	68.7	73.9	68.3	0.421
Current smokers, %	11.3	6.52	11.8	0.464
High education (≥11 years), %	37.3	39.1	37.1	0.874
Prevalent diabetes, %	10.5	15.2	10.1	0.285
Vitamin E supplementation, %	7.6	5.5	7.7	0.999
Physical Activity, MET-hour/week	89.8 (58.0, 130.5)	95.5 (64.8, 130.0)	88.3 (57.8, 131.1)	0.288
Alcohol consumption, g/day	9.91 (4.09, 19.2)	7.41 (3.14, 15.0)	10.2 (4.2, 19.8)	0.576
**Biochemical features**				
α-Tocopherol, μmol/L	31.5 (27.0, 37.1)	28.1 (24.0, 34.7)	31.8 (27.4, 37.4)	0.003
α-Tocopherol/cholesterol ratio, μmol/mmol	5.53 (4.88, 6.39)	5.24 (4.63, 5.73)	5.58 (4.91, 6.44)	0.015
γ-Tocopherol, μmol/L	1.34 (0.98, 1.79)	1.25 (0.88, 1.76)	1.35 (0.98, 1.79)	0.507
γ-Tocopherol/cholesterol ratio, μmol/mmol	0.24 (0.18, 0.31)	0.22 (0.19, 0.32)	0.24 (0.18, 0.31)	0.810
HbA1c, %	5.60 (5.40, 5.90)	5.80 (5.50, 5.90)	5.60 (5.40, 5.90)	0.088
HDL-cholesterol, mg/dL	62.5 (53.0, 77.0)	65.0 (54.0, 75.0)	62.0 (52.0, 78.0)	0.605
LDL-cholesterol, mg/dL	130.0 (108.0, 152.0)	125.0 (97.0, 152.0)	131.0 (108.0, 152.0)	0.237
Total cholesterol, mg/dL	220.0 (196.0, 249.0)	200.0 (185.0, 251.0)	221.0 (198.0, 248.0)	0.121
Triglycerides, mg/dL	105.0 (75.0, 138.0)	108.0 (72.0, 131.0)	104.5 (76.0, 138.5)	0.656

Values are presented as median and interquartile range or percentages (%). HDL: high density lipoproteins; LDL: low density lipoproteins; MET: metabolic equivalent. Differences in the characteristics of the participants with and without gallstone disease were tested for statistical significance using the chi-squared test or the Fisher’s exact test (when one of the expected values in one of the cells is less than 5) for categorical variables and the Wilcoxon’s rank-sum test for continuous variables.

**Table 2:** Odds ratio (OR) and 95% confidence interval (CI) for the association of α- and γ-tocopherol/cholesterol ratio with gallstone disease.

**Outcome**	**Tertiles of α-Tocopherol/Cholesterol Ratio**		
	**1**	**2**	**3**
Median α-tocopherol/cholesterol ratio (IQR), µmol/mmol	4.61 (4.24, 4.87)	5.52 (5.36, 5.72)	6.79 (6.38, 7.70)
Gallstones (yes/no) (46/536)	(21/173)	(18/176)	(7/187)
Age- and sex-adjusted OR (95% CI)	1.00 (reference)	0.89 (0.45–1.73)	0.32 (0.13–0.78)
Multivariable-adjusted OR (95% CI) *	1.00 (reference)	0.82 (0.42–1.63)	0.31 (0.13–0.76)
	**Tertiles of γ-Tocopherol/Cholesterol Ratio**		
	**1**	**2**	**3**
Median γ-tocopherol/cholesterol ratio (IQR), µmol/mmol	0.16 (0.13–0.18)	0.24 (0.22–0.26)	0.34 (0.31–0.41)
Gallstones (yes/no) (46/536)	(16/178)	(17/177)	(13/181)
Age- and sex-adjusted OR (95% CI)	1.00 (reference)	1.12 (0.54–2.29)	0.81 (0.38–1.74)
Multivariable-adjusted OR (95% CI) *	1.00 (reference)	1.08 (0.51–2.29)	0.77 (0.35–1.69)

IQR: Interquartile range; * Adjusted for age, sex, education, physical activity, smoking status, vitamin E supplementation, BMI, alcohol intake, and total energy intake.

## Supplementary Materials

The following are available online at http://www.mdpi.com/2072-6643/10/2/133/s1, Table S1: Sensitivity analyses: multivariable-adjusted odds ratio (OR) and confidence interval (CI) for the association of α- and γ-tocopherol/cholesterol ratio with gallstone disease after (a) excluding vitamin E supplement users, (b) excluding individuals with self-reported disease, (c) excluding individuals with type 2 diabetes, (d) modeling α- and γ-tocopherol without dividing them by cholesterol, and (e) performing a complete case analysis.
