# Supplementary material for: Association of Circulating Vitamin E (α- and γ-Tocopherol) Levels with Gallstone Disease
Source: Nutrients. 2018 Jan 27;10(2):133. doi: 10.3390/nu10020133 (PMC5852709; doi:10.3390/nu10020133)
Supplement: Supplementary file 1 [file nutrients-10-00133-s001.docx]

**Table S1**: Sensitivity analyses: Multivariable-adjusted Odds Ratio (OR) and Confidence Interval (CI) for the association of α- and γ-tocopherol/cholesterol ratio with gallstone disease after a) excluding vitamin E supplement users, b) excluding individuals with self-reported disease, c) excluding individuals with type 2 diabetes, d) modeling α- and γ-tocopherol without dividing them by cholesterol, and e) performing a complete-case analysis.

|  | **Tertiles of α-Tocopherol/Cholesterol Ratio** | | |
| --- | --- | --- | --- |
|  | **1** | **2** | **3** |
| a) excluding vitamin E supplement users (*n* = 44) * | 1.00 (Reference) | 0.71 (0.35–1.46) | 0.27 (0.10–0.69) |
| b) excluding individuals with self-reported disease (*n* = 30) ^†^ | 1.00 (Reference) | 0.86 (0.43–1.71) | 0.32 (0.13–0.80) |
| c) excluding individuals with type 2 diabetes (*n* = 61) ^†^ | 1.00 (Reference) | 0.81 (0.38–1.71) | 0.33 (0.13–0.87) |
| d) modeling α-tocopherol ^††^ | 1.00 (Reference) | 0.43 (0.12–0.89) | 0.43 (0.18–1.04) |
| e) performing a complete-case analysis (*n* = 558) ^†^ | 1.00 (Reference) | 0.69 (0.34–1.38) | 0.29 (0.12–0.72) |
|  | **Tertiles of γ-Tocopherol/Cholesterol Ratio** | | |
|  | **1** | **2** | **3** |
| a) excluding vitamin E supplement users (*n* = 44) ^*^ | 1.00 (Reference) | 1.14 (0.53–2.47) | 0.73 (0.32–1.67) |
| b) excluding individuals with self-reported disease (*n* = 30) ^†^ | 1.00 (Reference) | 1.04 (0.49–2.20) | 0.71 (0.31–1.60) |
| c) excluding individuals with type 2 diabetes (*n* = 61) ^†^ | 1.00 (Reference) | 1.13 (0.50–2.58) | 0.87 (0.37–2.05) |
| d) modeling γ-tocopherol ^††^ | 1.00 (Reference) | 1.06 (0.49–2.38) | 0.90 (0.40–2.02) |
| e) performing a complete-case analysis (*n* = 558) ^†^ | 1.00 (Reference) | 1.14 (0.54–2.41) | 0.64 (0.28–1.46) |

* Adjusted for age, sex, education, physical activity, smoking status, BMI, alcohol intake, and total energy intake; ^†^ Adjusted for age, sex, education, physical activity, smoking status, BMI, alcohol intake, total energy intake, and vitamin E supplementation; ^††^ Adjusted for age, sex, education, physical activity, smoking status, BMI, alcohol intake, total energy intake, vitamin E supplementation, and cholesterol.
